# Navigating the Spectrum of Cariology: Laser-Mediated Theranostics in Pediatric and Adult Populations

**DOI:** 10.7759/cureus.113613

**Published:** 2026-07-29

**Authors:** Joanith Medina Barreto, Giuliana Bazalar, Grisel J Garcia, Dayana Paucarima Mejia, Dayana Gutierrez Monterrosa, Vanessa Serna Sierra, Luisana S Rodriguez

**Affiliations:** 1 Dentistry, University of Carabobo, Valencia, VEN; 2 Dentistry, Universidad Alas Peruanas, Lima, PER; 3 Dentistry, Ciencias Medicas Medicas Zoilo Marinello, Marinello, CUB; 4 Dentistry, Universidad Regional Autónoma de Los Andes, Ambato, ECU; 5 Dentistry, Universidad del Magdalena, Santa Marta, COL; 6 Dentistry, Universidad Cooperativa de Colombia, Medellin, COL; 7 Dentistry, Universidad de Carabobo, Valencia, VEN; 8 General Dentistry, Boston University School of Medicine, Boston, USA

**Keywords:** er:yag laser, laser-induced fluorescence, minimally invasive dentistry, patient-centered, dental caries

## Abstract

Laser technology has broadened the scope of dental caries management, supporting diagnosis, prevention, and treatment within a single minimally invasive framework, but its performance depends on operator expertise and on parameters matched to the tissue and the patient. This narrative review synthesizes contemporary evidence on laser-mediated theranostics in pediatric and adult cariology, comparing age-specific diagnostic and therapeutic applications and their impact on patient-centered, minimally invasive care. Peer-reviewed systematic reviews, randomized controlled trials, and observational studies from the last five years were identified in PubMed and Scopus. Across the evidence, laser-assisted approaches offer distinct benefits in each population. In primary dentition, laser fluorescence improves the detection of early enamel demineralization when dentition-specific thresholds are applied, while erbium-doped yttrium aluminum garnet (Er:YAG) systems reduce pain, anxiety, and the need for local anesthesia, improving cooperation. In permanent dentition, erbium lasers support conservative management of secondary caries, decontamination of affected tissue, and preservation of existing restorations. Antimicrobial photodynamic therapy (aPDT) consistently reduces cariogenic biofilms in both populations, with particular value in adults presenting more complex periodontal and restorative conditions. Taken together, the evidence supports tailoring laser-assisted diagnosis and treatment to dentition type, lesion characteristics, and clinical objectives rather than relying on a single standardized approach; laser therapy is an effective, conservative option for caries management in both dentitions that improves patient comfort, although standardized clinical protocols are still needed.

## Introduction and background

Dental caries remains one of the most prevalent chronic conditions worldwide and is now understood as a diet- and biofilm-driven non-communicable disease rather than a simple infection, which reframes its prevention and management around ecological and behavioral control as much as operative repair [1]. Within this model, the appeal of laser technology is that it spans the whole spectrum of care, supporting diagnosis, prevention, and treatment while remaining minimally invasive and, in many cases, more comfortable for the patient [2].

The organizing concept of this review is theranostics. The term combines therapeutics and diagnostics and describes the use of a single technology or a single family of technologies to both detect and treat disease. Applied to cariology, a theranostic approach means that the same laser platform can quantify early mineral loss at low power and, at high power, selectively remove carious tissue, so that detection, prevention, and intervention are delivered within one conservative philosophy rather than as separate steps [2,3].

A comparison between pediatric and adult populations is clinically important because laser-assisted care is not a single standardized intervention. Enamel maturity, lesion depth, pulpal status, biofilm complexity, and patient behavior all differ between primary and permanent dentition, so diagnostic thresholds and treatment parameters validated in one context may not transfer to the other; primary enamel is thinner, less mineralized, and more porous, and primary teeth have proportionally larger pulp chambers, which alters both fluorescence readings and safe ablation parameters [4,5]. Clinical priorities also diverge. In children, comfort and fear reduction determine cooperation and retention in preventive care, whereas in adults, the dominant concern is the long-term maintenance of increasingly complex restorative work. Treating these two populations as one obscures both sets of requirements.

This review therefore synthesizes contemporary evidence on laser-mediated theranostics across the lifespan. It is organized into six themes: pediatric management and the reduction of dental anxiety; adult management of secondary caries and restoration longevity; diagnostic differences arising from enamel morphology; biofilm control through antimicrobial photodynamic therapy (aPDT) as common ground between the populations; the patient journey and patient-centered outcomes; and a synthesis toward dentition-specific protocols, including current limitations and research priorities.

## Review

Methodology

This is a narrative review and was not conducted or reported as a systematic review. Searches were performed in PubMed/MEDLINE and Scopus for records published between January 2020 and April 2026, with seminal older publications retained where they remain the primary source for an established concept, such as device threshold criteria. Search terms combined controlled vocabulary and free-text terms across three concept blocks: intervention (laser, erbium-doped yttrium aluminum garnet (Er:YAG), erbium, chromium-doped yttrium scandium gallium garnet (Er,Cr:YSGG), neodymium-doped yttrium aluminum garnet (Nd:YAG), carbon dioxide laser, photobiomodulation, laser fluorescence, and antimicrobial photodynamic therapy), condition (dental caries, cariology, secondary caries, demineralization, and dental biofilm), and population (pediatric, children, primary dentition, deciduous teeth, adult, and permanent dentition). Reference lists of retrieved articles and relevant consensus reports were hand-searched for additional records.

Eligible publications were peer-reviewed systematic reviews, meta-analyses, randomized controlled trials, clinical and observational studies, and international consensus reports, published in English, that reported diagnostic or therapeutic applications of laser technologies in caries management in either dentition. Records were excluded when they addressed laser applications outside cariology, such as purely soft tissue surgical or implant procedures; when they were case reports, editorials, or commentary without primary or synthesized data; or when insufficient methodological detail prevented interpretation. Records were screened by title and abstract, and full texts of potentially relevant articles were assessed against these criteria, with disagreements resolved by discussion among the authors.

Evidence was synthesized narratively and thematically around the six themes listed above. Consistent with a narrative design, no PRISMA protocol, formal risk-of-bias instrument, or certainty-of-evidence appraisal was used, and no quantitative synthesis, such as a meta-analysis or meta-regression, was undertaken. The findings should therefore be read as an interpretive synthesis rather than as a pooled estimate of effect.

Pediatric management: dental anxiety and the needle-free goal

Pediatric laser-mediated cariology should be understood not only as a restorative technique but also as a behavioral strategy to reduce fear, pain, and avoidance of dental care. In children, dental anxiety is frequently intensified by the sound and vibration of rotary handpieces, the sensation of pressure during cavity preparation, and the anticipation of local anesthesia. The needle-free goal, therefore, does not imply eliminating anesthesia in every case, but rather minimizing injections whenever lesion depth, pulpal status, and patient safety allow it.

Initial assessment should include caries risk, lesion status, and dentition type. In primary dentition, thinner enamel, larger pulp chambers, and faster lesion progression require conservative parameters and careful thermal control. Low-power diagnostic lasers may support early detection of enamel demineralization, allowing preventive intervention before cavitation. When operative treatment is required, Er:YAG laser systems offer an alternative to conventional turbines by selectively ablating carious tissue through water-mediated microexplosions, reducing vibration and mechanical pressure. Recent clinical evidence shows that Er:YAG laser caries removal in children is associated with lower pain perception, reduced anxiety, improved cooperation, and decreased need for local anesthesia compared with conventional rotary instrumentation [6,7].

This psychological benefit is central to pediatric management. A child who experiences restorative care as quieter, less painful, and less threatening may be more likely to accept future appointments, improving longitudinal compliance and retention in preventive programs. A recent systematic review comparing Er:YAG laser and dental turbines in pediatric dentistry reported that laser treatment resulted in less pain and greater comfort, although the procedure duration may be longer than with conventional treatment [6]. Similarly, a randomized clinical trial in deciduous teeth found that Er:YAG laser preparation reduced pain and the need for local anesthesia while preserving pulp vitality [8], and combining laser treatment with structured behavior management techniques has been proposed as a way to reduce procedural anxiety further [9].

Laser-assisted approaches also fit within the theranostic concept because they support diagnosis, prevention, and treatment under a single minimally invasive philosophy. Low-power fluorescence-based methods help identify early mineral changes, while high-power erbium lasers assist in selective caries removal and cavity preparation. This is especially relevant in primary teeth, where preserving sound tissue and protecting the pulp are essential. Reviews on pediatric laser dentistry emphasize that lasers may improve patient comfort, reduce intraoperative anxiety, and support clinical cooperation when appropriately selected and calibrated [3].

The anxiety-reduction effect is not limited to hard tissue caries removal. Low-level laser stimulation and laser acupuncture have also been investigated as adjunctive methods to reduce pain and anxiety during pediatric dental procedures, including local anesthesia administration [10-13]. These approaches sit alongside the broader repertoire of non-pharmacological behavior management and, at the more severe end of the spectrum, treatment under general anesthesia, both of which remain relevant to the anxious pediatric patient [14,15].

Clinically, laser use should be individualized. Early non-cavitated lesions may be managed through diagnosis, remineralization, and monitoring. Shallow or moderate cavitated lesions in cooperative children may benefit from Er:YAG laser preparation to reduce pain, noise, vibration, and anesthesia requirements. Deep lesions, irreversible pulpal symptoms, or uncooperative behavior may still require conventional anesthesia or alternative behavior management.

Adult management: secondary caries, periodontal health, and restoration longevity

Secondary caries is one of the leading causes of restoration failure and develops at the margins of restorations. Its biological origin is similar to that of primary caries, as both result from biofilm accumulation, and it may be associated with poorly adapted restorations, marginal defects, or pre-existing carious lesions. Diagnosis combines laser fluorescence, clinical examination, and radiographic assessment, while management consists of repairing or replacing the restoration depending on lesion size and restoration longevity [16,17].

Secondary caries is particularly relevant around crowns and fixed dental bridges, where connectors, the space beneath the pontic, and inadequate marginal adaptation promote plaque accumulation, increasing the risk of secondary caries and periodontal disease. Proper oral hygiene and the correct use of hygiene aids are therefore essential to prolong the survival of restorations. In a seven-year study of 423 patients with fixed prostheses, 69% of restorations remained successful, largely because of good oral hygiene and a lower incidence of secondary caries [18].

The Er:YAG laser represents a conservative alternative for removing secondary caries around fixed restorations while preserving both tooth structure and restorative materials. Its microexplosions remove carious tissue in a minimally invasive manner, reducing vibration, noise, and pain, while also providing bactericidal and bacteriostatic effects. One study demonstrated complete removal of carious lesions and a reduction in microbial load, which is particularly beneficial for uncooperative, anxious, pediatric, and orthodontic patients [19].

Lithium disilicate and zirconia crowns and bridges fabricated using computer-aided design and computer-aided manufacturing (CAD/CAM) systems provide improved precision and adhesion. However, when recurrent caries develops, removing these restorations with rotary instruments may damage both the restoration and the underlying tooth. In this context, the Er:YAG laser allows removal of crowns, brackets, resin restorations, and veneers without affecting the restoration, dentin, or pulp. In vitro studies have shown that lithium disilicate and zirconia monolithic crowns can be retrieved and reused, although zirconia crowns required longer debonding times [20,21]. These findings derive from laboratory models rather than clinical trials and should be interpreted accordingly.

A recent review highlighted that different laser systems have specific applications in prosthodontics. The erbium laser is particularly effective for minimally invasive removal of carious tissue and restorative materials while preserving tooth structure and enhancing the adhesion of prosthetic materials; it also reduces patient stress and surgical bleeding, although appropriate eye protection, careful patient selection, and professional training are required [22]. A systematic review of studies published between 2017 and 2022 concluded that erbium lasers (Er:YAG and Er,Cr:YSGG) are effective for caries removal and restorative procedures, whereas the diode laser performed better in soft tissue applications [23]. A further systematic review comparing erbium systems found that Er:YAG produced more consistent results for caries removal and surface preparation, whereas Er,Cr:YSGG showed lower efficacy and Nd:YAG the lowest efficiency for hard tissue ablation, again with a call for additional clinical studies [24].

In summary, the available evidence indicates that the Er:YAG laser is an effective option for managing secondary caries around crowns and fixed dental bridges. Its ability to remove carious tissue and debond restorations without damaging either the restorative material or the tooth structure may contribute to restoration longevity, although further clinical studies are needed to strengthen the evidence and expand its application in prosthodontics.

Diagnostic differences: morphological variation between primary and permanent enamel

The interpretation of laser fluorescence in caries detection is strongly influenced by structural differences between primary and permanent enamel, since these variations directly affect how light interacts with the dental tissues and how diagnostic signals are generated and interpreted [4,25,26]. In primary dentition, enamel presents reduced thickness, lower mineralization, and greater porosity, which influence its optical behavior and fluorescence response compared with permanent enamel. These differences can result in higher fluorescence readings even at early stages of mineral loss, which may affect the interpretation of lesion severity in clinical practice [4,27].

Permanent enamel, in contrast, is more mature and densely mineralized, with a more organized prism structure and lower permeability. This leads to a more stable interaction with laser light and, under comparable clinical conditions, generally lower fluorescence values. In practical terms, identical fluorescence readings may not reflect the same histopathological stage across dentitions, an important consideration when interpreting diagnostic outputs in both pediatric and adult patients [4,25].

Because of these structural and optical differences, laser fluorescence devices such as DIAGNOdent do not perform uniformly across dentitions [4,26]. Evidence indicates that diagnostic performance varies with enamel characteristics and that interpretation should not rely on a single universal threshold; dentition-specific calibration is required to reduce false-positive and false-negative results, particularly in early enamel lesions [26,28]. The threshold values most widely used in practice were largely derived from permanent teeth, and their direct transfer to primary teeth is a recognized source of misclassification [29]. The principal structural, optical, and threshold-related differences between the two dentitions are summarized in Table 1.

**Table 1:** Structural, optical, and threshold-related differences influencing laser fluorescence interpretation in primary versus permanent dentition ICDAS: International Caries Detection and Assessment System Cut-off values reflect criteria most commonly cited for the 655 nm laser fluorescence device and vary between devices, tooth surfaces, and studies. Sources: [4,26,29,30]

Parameter	Primary dentition	Permanent dentition
Enamel structure	Thinner, less mineralized, more porous, with less organized prism arrangement	Thicker, highly mineralized, organized prism structure with lower permeability
Optical behavior	Greater light scattering; comparatively higher fluorescence readings at equivalent mineral loss	More stable interaction with laser light; comparatively lower baseline values
Commonly applied laser fluorescence cut-offs (655 nm)	Manufacturer thresholds were derived largely from permanent teeth; lower, study-specific cut-offs have been reported as necessary and require local validation	Sound 0-14; enamel caries 15-20; dentinal caries 21 or above; operative intervention commonly considered above 30
Diagnostic performance	Weakest at the early enamel threshold; more reliable at the dentinal threshold	Acceptable at the dentinal threshold; limited sensitivity for early enamel lesions
Principal risk of misclassification	False-positive readings and overestimation of lesion severity	False-negative readings in early, non-cavitated enamel lesions
Clinical implication	Dentition-specific calibration essential; use strictly as an adjunct to ICDAS and radiographic assessment	Manufacturer thresholds broadly applicable but still adjunctive to visual and radiographic criteria

Laser fluorescence should therefore not be used as a standalone diagnostic method. Its accuracy improves significantly when combined with validated systems such as the International Caries Detection and Assessment System (ICDAS) and, when necessary, radiographic evaluation [4,25,30]. Fluorescence-based measurement is also subject to well-recognized confounders: dental plaque, extrinsic stains, calculus, prophylaxis paste residues, hypomineralization defects, and existing restorative materials can all elevate readings independently of demineralization, so surfaces should be cleaned and dried and readings interpreted against clinical findings rather than in isolation [4,29]. Recognizing the biological and optical differences between primary and permanent dentition, together with these technical limitations, is essential for improving diagnostic reliability across age groups. Because caries is fundamentally a biofilm-driven disease, accurate interpretation of fluorescence-based findings must ultimately be understood within the broader biological context of microbial activity.

Biofilm management: antimicrobial photodynamic therapy as common ground

The oral cavity constitutes a complex, dynamic microbial ecosystem. Bacteria such as *Streptococcus mutans*, *Porphyromonas gingivalis*, and *Fusobacterium nucleatum* initiate biofilm formation by adhering to the enamel surface through the acquired salivary pellicle, a protein-rich layer that allows additional microorganisms to accumulate into a highly organized community. Dental biofilm exhibits a diverse microbial composition within a three-dimensional architecture, embedded in an extracellular matrix of polysaccharides, peptides, and nucleic acids, and adheres to enamel, root cementum, and implant surfaces, creating protected microenvironments that favor bacterial survival. With frequent sugar exposure, acidogenic bacteria lower the local pH, leading to enamel demineralization and caries, while dysbiotic changes within the biofilm also promote inflammatory processes such as periodontal disease [31,32]. Reduced salivary flow and inadequate daily mechanical biofilm removal further accelerate biofilm maturation, so caries control depends on preventing bacterial activity, interrupting the ecological factors that promote cariogenic biofilm, and promoting remineralization [1].

Within this context, antimicrobial photodynamic therapy (aPDT) has emerged as a strategy to overcome the intrinsic resistance of mature biofilms. aPDT combines a photosensitizer with laser light of a specific wavelength, activating molecular oxygen to generate reactive oxygen species and highly unstable free radicals. Because of their high chemical reactivity, these species rapidly oxidize bacterial proteins, lipids, and nucleic acids, producing irreversible cellular damage while disrupting the structural integrity of the biofilm [33,34].

This mechanism is particularly relevant in pediatric dentistry, where early childhood caries is primarily driven by aciduric biofilms on relatively accessible tooth surfaces. By penetrating these biofilms, suppressing bacterial colonization, and preventing new biofilm formation, photosensitizer-mediated aPDT can reduce both the incidence and severity of carious lesions while preserving surrounding healthy tissue, and its non-invasive, painless nature makes it a valuable adjunctive therapy for children [35]. It should be noted that much of the mechanistic and photosensitizer development evidence in this area derives from in vitro biofilm models and animal studies rather than from clinical trials in children [33-35].

In contrast, the clinical need for aPDT increases in adults, where biofilms are more mature and frequently develop within deep periodontal pockets, root caries, and restorative margins, which hinder complete mechanical debridement, and where complex root anatomy favors bacterial persistence and recolonization despite scaling and root planing. As an adjunctive therapy, aPDT disrupts mature biofilms by eliminating early and late colonizers such as *S. mutans*, *Fusobacterium nucleatum*, *Porphyromonas gingivalis*, and *Aggregatibacter actinomycetemcomitans*, thereby improving periodontal outcomes, particularly in deep defects; combined with methylene blue, aPDT has also demonstrated the ability to destroy *Enterococcus faecalis*, a principal microorganism associated with endodontic treatment failure [36,37]. Although clinical complexity determines how aPDT is implemented in each population, its therapeutic objective remains identical: to reduce the burden of acidogenic and pathogenic bacteria through minimally invasive means.

The patient journey: comparing laser-based interventions across the lifespan

Laser-based interventions have extended treatment goals beyond disease management to encompass the overall patient experience across the continuum of care. The biological and behavioral differences discussed above also shape distinct patient journeys, so patient-centered care requires not only selecting the appropriate technique but also adapting therapeutic objectives to the patient's stage of life. In pediatric dentistry, laser applications primarily support preventive, minimally invasive strategies focused on preserving healthy tooth structure and reducing future caries risk, whereas adult care increasingly emphasizes conservative rehabilitation through the management of secondary caries, the preservation of existing restorations, and the long-term maintenance of oral function [2].

The patient journey begins with treatment acceptance, where behavioral factors strongly influence outcomes. Randomized trials have shown that Er:YAG laser-assisted caries removal significantly reduces pain perception, decreases the need for local anesthesia, and improves cooperation compared with conventional rotary instrumentation [7,38]. A recent meta-analysis reinforced that laser-assisted caries removal consistently reduces pain and anesthesia requirements while maintaining comparable pulp vitality and restoration survival, despite longer operative times [39]. Such characteristics are particularly valuable during childhood, when positive dental experiences may foster lifelong adherence to preventive care.

As patients transition into adulthood, therapeutic priorities shift progressively from prevention toward preservation and rehabilitation. Beyond clinical effectiveness, photobiomodulation reduces postoperative pain, inflammation, and discomfort, improving oral health-related quality of life across multiple procedures [40]. Accordingly, patient-reported outcome measures, including oral health-related quality of life, are increasingly recognized as essential indicators of treatment success, complementing conventional clinical parameters [40,41].

The post-treatment phase further illustrates these age-specific differences. In pediatric patients, follow-up emphasizes reinforcement of preventive behaviors, parental engagement, and maintenance of caries-free dentitions through minimally invasive strategies, including laser-assisted caries prevention and pit-and-fissure management [42]. Conversely, long-term adult care prioritizes restoration longevity, prevention of recurrent disease, and sustained function through conservative rehabilitative protocols [2]. Across both populations, laser-based interventions may enhance adherence to maintenance care by reducing discomfort and promoting more positive perceptions of dental treatment.

Synthesis: toward dentition-specific clinical protocols

The use of lasers as an alternative to conventional methods for caries removal is becoming more common, as is their use for caries detection and prevention [24,39]. Specifically for caries removal, the most commonly used lasers are Er:YAG, Er,Cr:YSGG, and Nd:YAG [24]. These options can reduce intraoperative anxiety, increase cooperation in children, lessen pain, and improve comfort; however, treatment duration and the establishment of specific protocols according to laser type and target tissue still require refinement [24,38].

Although operator expertise is important, other factors are crucial, such as the composition of the tissue to be treated. Carious tissue contains more water than healthy tissue, so the laser ablation rate in dentin may be higher than in enamel [8]. The Er:YAG laser shows a high affinity for water, making it well-suited to cavity preparation in both enamel and dentin. The wavelength is critical because high absorption in dentin and enamel is required for efficient, biologically safe ablation [2]. Other factors, including pulse width, pulse repetition rate, power density, the tissue's thermal resting state, and the delivery method, strongly influence the rate of hard tissue ablation [43].

Diagnosis is best performed with a low-power laser, whereas caries removal is performed with a high-power laser. Laser fluorescence can improve the precision and speed of early caries detection compared with visual evaluation or radiographs [5,29]. Visual examination has high specificity but low sensitivity and reproducibility, whereas radiographs detect demineralization limited to dentin rather than enamel [29]. Quantitative light-induced fluorescence devices are effective for measuring incipient demineralization in primary teeth, thereby enabling preventive treatments, such as topical fluoride application, before cavitation [5]. In permanent teeth, red laser light at 655 nm (DIAGNOdent) is used to assess fluorescence from porphyrins, and a score above 30 is commonly considered the threshold for initiating operative treatment [30].

For treatment, the erbium laser is the reference standard. Its mechanism relies on absorption of light by water in the infected tissue, producing microexplosions that dissolve carious tissue while preserving sound structure [8]. In primary teeth, lower power settings (approximately 120-150 mJ) are used to protect the pulp from heat [8]. Bringing these threads together, laser diagnosis, prevention, and treatment increasingly justify a bifurcated, dentition-specific approach, and a global consensus report of the World Federation for Laser Dentistry provides a framework for laser-assisted caries treatment and prevention that can anchor such protocols [44]. Representative laser systems and their principal characteristics are summarized in Table 2.

**Table 2:** Principal laser systems used in cariology and their characteristics relevant to clinical decision-making Er:YAG: erbium-doped yttrium aluminum garnet, Er,Cr:YSGG: erbium, chromium-doped yttrium scandium gallium garnet, Nd:YAG: neodymium-doped yttrium aluminum garnet, CO2: carbon dioxide Sources: [8,30,44]

Feature	Er:YAG	Er,Cr:YSGG	Nd:YAG	CO2 laser
Type	Pulsed	Pulsed	Pulsed	Pulsed or continuous wave
Wavelength	2940 nm	2780 nm	1064 nm	9600 nm
Spectrum	Infrared	Infrared	Near-infrared	Infrared
Main indication	Hard tissue	Hard and soft tissue	Lower effectiveness in hard tissue	Hard tissue
Key characteristics	Remineralizing potential, selective ablation of carious tissue, smear-layer removal, and improved adhesive procedures	Clean tissue cuts, minimally invasive, enamel etching, and caries removal	Better as an adjunct for disinfection or surface modification than for caries removal	Smooth cavity margins with effective ablation efficiency

Limitations, cost, and implementation considerations

Several constraints temper the enthusiasm for laser-mediated cariology and should be weighed before adoption. The first is economic. Erbium laser platforms represent a substantial capital investment relative to conventional rotary equipment, and this cost is compounded by maintenance, handpiece and tip replacement, and the additional chair time repeatedly documented for laser preparation compared with rotary instrumentation [6,24,39]. Because reimbursement rarely distinguishes laser-assisted from conventional restorations, the return on that investment depends on case volume and case mix rather than on procedure-level fees, and formal cost-effectiveness analyses comparing laser-assisted with conventional caries management remain scarce. Feasibility therefore differs sharply between well-resourced private practices and public or community services, and availability remains uneven across health systems, which limits the generalizability of the evidence reviewed here.

The second constraint is training and safety. Laser-assisted treatment is highly technique-sensitive: outcomes depend on the correct selection of wavelength, energy per pulse, repetition rate, water cooling, and working distance; inadequate parameters risk thermal injury to the pulp or surrounding tissues. Structured training, certification, mandatory wavelength-specific eye protection for the patient and dental team, and careful case selection are prerequisites rather than refinements [22,43]. Contraindications and cautions also apply, including lesions in close proximity to the pulp, where thermal risk is elevated; patients who cannot tolerate the procedure without sedation or anesthesia, regardless of technique; sites where access or visibility prevents controlled irradiation; and, for aPDT, known sensitivity to the photosensitizing agent. Erbium wavelengths are additionally unsuited to certain restorative materials, and laser removal of existing restorations requires knowledge of how each material responds.

The third constraint concerns the nature of the evidence itself. A substantial proportion of the supporting data, particularly for crown retrieval, surface modification, photosensitizer development, and biofilm eradication, comes from in vitro and ex vivo models that cannot replicate the oral conditions of salivary flow, thermal exposure, and behavioral activity [20,21,33-35]. Clinical evidence, where it exists, is dominated by small single-center trials with short follow-up and heterogeneous outcome definitions, and both recent systematic reviews and a meta-analysis explicitly attribute their inability to pool results to this heterogeneity [6,24,39]. The consequence is the recurring conclusion of this literature: there is still no standardized, internationally agreed protocol specifying laser parameters by dentition, tissue, and lesion depth, and the recent consensus report of the World Federation for Laser Dentistry represents an important first step rather than a settled standard [44]. Laboratory findings should accordingly be read as mechanistic support, not as clinical endorsement.

Future research priorities

Four priorities follow from these limitations. First, adequately powered multicenter randomized controlled trials are needed, with standardized reporting of laser parameters, blinded outcome assessment where feasible, and long-term follow-up to capture restoration survival, secondary caries, and pulp vitality rather than only intraoperative comfort. Second, dentition-specific validation studies should establish and independently confirm laser fluorescence thresholds for primary teeth, since current cutoffs largely derive from the permanent dentition and are a documented source of misclassification [26,29]. Third, formal economic evaluation is required: cost-effectiveness and cost-utility analyses that incorporate equipment cost, chair time, training, and long-term restoration survival would allow rational decisions about adoption in different practice settings. Fourth, consensus-based clinical guidelines should build on existing international frameworks to specify parameters by dentition, tissue, and lesion depth, supported by core outcome sets so that future trials can be pooled [44]. Emerging directions, including artificial intelligence-assisted interpretation of fluorescence and imaging data and the integration of laser diagnostics into digital caries monitoring workflows, warrant evaluation alongside these priorities, provided such tools are validated prospectively before clinical deployment.

## Conclusions

Laser-mediated theranostics offers an effective and conservative approach to caries management across the lifespan, uniting diagnosis, prevention, and treatment within a single minimally invasive philosophy. In pediatric care, its greatest value lies in reducing pain, anxiety, and the need for local anesthesia, thereby supporting cooperation and long-term adherence, whereas in adults, it enables conservative management of secondary caries and the preservation of complex restorations. Laser fluorescence improves early lesion detection when interpreted with dentition-specific thresholds and alongside validated visual and radiographic criteria, and antimicrobial photodynamic therapy provides a shared biofilm control strategy whose clinical need grows with the anatomical complexity seen in adults. These benefits must be balanced against equipment cost, uneven availability, mandatory operator training, defined contraindications, longer operative times, and an evidence base still weighted toward laboratory models and small single-center trials. The recurring limitation is the absence of universal protocols: tissue characteristics, caries risk, dentition type, and laser parameters must be individualized. Multicenter randomized trials, dentition-specific threshold validation, formal economic evaluation, and consensus-based guidelines are therefore needed before laser-mediated theranostics can be adopted uniformly, but the current evidence already supports its role as a patient-centered, minimally invasive option in both primary and permanent dentitions.
